# 

**Published:** 1946

**Authors:** 


**NOTICE**

**Owing to the great increase in the cost of production, it has become necessary to raise the price of the Journal from Three Shillings to Four Shillings for each issue. Annual subscription, Sixteen Shillings.**

### The Medical Library of the University of Bristol

WITH WHICH IS INCORPORATED

**The Library of the Bristol Medico-Chirurgical Society**

---

*The following donations have been received since the publication of the list in the last issue.*

| | | |
|---|---|---|
| Dame Katharine Furse (1) | .. .. .. .. | 1 Volume. |
| Michell Clarke Fund (2) .. | .. .. .. .. | 21 Volumes. |
| National Foundation for Infantile Paralysis (3) | .. | 1 Volume. |
| J. E. Shaw Fund (4) | .. .. .. .. .. | 2 Volumes. |
| William R. Warner and Co. Ltd. (5) .. | .. .. | 1 Volume. |
| Wilmer Ophthalmological Institute (6) | .. .. | 1 Volume. |

Unbound periodicals have also been received from Professor C· Bruce Perry and Messrs. John Wright and Sons Ltd.

### THE ONE HUNDRED AND EIGHTY FIRST LIST OF BOOKS.

The figures in round brackets refer to the figures after the names of the donors. The books to which no such figures have been attached have either been bought from the Library Fund or received through the *Journal.*

| | | | | |
|---|---|---|---|---|
| **Aitken, R.** | *The Problem of Lupus Vulgaris* | | (2) | 1946 |
| **Armstrong, J. R.** | *Bone-grafting in the Treatment of Fractures* | | | 1945 |
| **Brain, W. R., and Strauss, E. B.** | *Recent Advances in Neurology and Neuropsychiatry* | 5th Ed. | (2) | 1946 |
| **Broster, L. R.** | *Endocrine Man* | | | 1945 |
| **Browne, F. J.** | *Antenatal and Postnatal Care* | 6th. Ed. | (2) | 1946 |
| **Bulbulian, A. H.** | *Facial Prosthesis* | | | 1945 |
| **Cowdry, E. V.** | *Textbook of Histology* | 3rd Ed. | | 1944 |
| **Ellis, Havelock** | *Studies in the Psychology of Sex* | Vol. 2 | (1) | 1921 |
| **Fishbein, M.(Ed.)** | *A Bibliography of Infantile Paralysis* 1789–1944 | | (3) | 1946 |
| **Fleming, A. (Ed.)** | *Penicillin* | | (2) | 1946 |
| **Gantier, R.** | *Health Organisation and Biological Standardisation* | | (2) | 1946 |
| **Goldring, W. and Chasis, H.** | *Hypertension and Hypertensive Disease* | | (2) | 1944 |
| **Henderson, D. K. and Gillespie, R. D.** | *Textbook of Psychiatry* | 6th. Ed. | | 1944 |
| **Hertz, J.** | *On Goitre and Allied Diseases* | | (2) | 1943 |
| **Holmes, G.** | *Introduction to Clinical Neurology* | | (2) | 1946 |
| **Horder, Lord (Ed.)** | *British Encyclopædia of Medical Practice* :— | | | |
| | (*a*) *Cumulative Supplement* | | (4) | 1946 |
| | (*b*) *Medical Progress* | | (4) | 1946 |
| **Hueper, W. C.** | *Occupational Tumors and Allied Diseases* | | (5) | 1942 |
| **Johnstone, R. W.** | *Textbook of Midwifery* | 12th Ed. | (2) | 1945 |

| | | | |
|---|---|---|---|
| **Kerr, D. J. A.** | *Forensic Medicine* | 4th Ed. | 1946 |
| **Levy, I. R.** | *A Textbook for Dental Assistants* | | 1942 |
| **Lewis, Sir T.** | *Research in Medicine and other Addresses* | 2nd Ed. | 1945 |
| **McDowall, R. J. S.** | *Handbook of Physiology and Biochemistry* | 38th Ed. | 1944 |
| **Martin, M.** | *Make Yours a Fine Baby* | (2) | 1945 |
| **Merritt, A. H.** | *Periodontal Diseases* | 3rd Ed. (2) | 1945 |
| **Newman, C.** | *Medical Emergencies* | 3rd Ed. | 1946 |
| **Novak, E.** | *Textbook of Gynæcology* | 2nd Ed. | 1944 |
| **Osler, W.** | *Principles and Practice of Medicine* | 15th Ed. (2) | 1944 |
| **Paterson, R., Tod, M., and Russell, M.** | *Results of Radium and X-ray Therapy in Malignant Disease* | (2) | 1946 |
| **Quigley, T. B.** | *Plaster of Paris Technique* | | 1945 |
| **Richards, R. L.** | *Peripheral Circulation in Health and Disease* | (2) | 1946 |
| **Rosendal, T.** | *The Conducting Properties of the Human Organism to Alternating Current* | (2) | 1940 |
| **Salzmann, J. A.** | *Principles of Orthodontics* | | 1943 |
| **Semon, H. C. G., and Moritz, A.** | *Atlas of the Commoner Skin Diseases* | 3rd Ed. | 1946 |
| **Seth, G. and Guthrie, D.** | *Speech in Childhood* | | 1935 |
| *Sex in Marriage* (Marriage Guidance Council) | | | 1946 |
| **Smillie, I. S.** | *Injuries of the Knee Joint* | (2) | 1946 |
| **Smithers, D. W.** | X-*ray Treatment of Accessible Cancer* | (2) | 1946 |
| **Tanner, W. E.** | *Sir W. Arbuthnot Lane* | | 1946 |
| **Todd, A. T.** | *Medical Aspects of Growing Old* | | 1949 |
| **Vannotti, A., and Delachaux, A.** | *Der Eisenstoffwechsel und seine klinische Bedeutung* | (2) | 1942 |
| **Weber, F. P.** | *Rare Diseases and some Debatable Subjects* | (2) | 1946 |
| **Widdowson, T. W.** | *Special or Dental Anatomy and Physiology* | Vol. 1 7th Ed. | 1946 |

TRANSACTIONS, REPORTS, JOURNALS, ETC.

| | | |
|---|---|---|
| Collected Reprints : Wilmer Ophthalmological Institute. Vol. 6 | (6) | 1942 |
| Modern Treatment Yearbook | (2) | 1945 |
| Quarterly Cumulative Index Medicus. Vol. 38. July to December | | 1945 |
| Yearbook of the Eye, Ear, Nose and Throat | (2) | 1945 |

### Publications Received

From OXFORD UNIVERSITY PRESS :

*The Anatomy of the Bronchial Tree.* By R. C. BROCK. 1946. Price 42s.

*The Nervous Child.* By HECTOR CHARLES CAMERON. Fifth Edition. 1946. Price 10s. 6d.

From H. K. LEWIS & CO. LTD.

*A Handbook of Radiography.* By JOHN A. ROSS. Second Edition. 1946. Price 10s. 6d.

From JOHN WRIGHT & SONS LTD.

*An Atlas of the Commoner Skin Diseases.* By HENRY C. G. SEMON AND ARNOLD MORITZ. Third Edition. 1946. Price 50s. Net.

*Demonstrations of Physical Signs in Clinical Surgery.* By HAMILTON BAILEY. Tenth Edition. 1946. Price 30s.

*British Journal of Surgery.* July, 1946. Price 12s. 6d. Yearly Subscription 42s.

### Local Medical Notes

**Royal College of Obstetricians and Gynæcologists.**—Dr. Mabel Potter.

EXAMINATION RESULTS.

Students of the University have recently passed the following examinations :—

**University of Bristol.**—M.D.—J. J. Kempton.

M.B., Ch.B.—*First Examination :* A. M. ApSimon, K. B. Arney, Margaret M. Ashton, Pearl D. Barker-Wyatt, Ann E. Boulter, R. B. Brodie, June M. Butler, Nancy C. C. Carter, G. V. Catford, Jean M. Elliott, V. Gavrilovic, W. E. L. Gordon, M. Gummer, Ann M. Haines, G. J. Hillier, Audrey V. Hooper, Elisabeth Hopkins, R. J. Hunt, R. A. Iles, D. J. A. Jarvis, June P. Lawson, June K. Lloyd, D. G. MacLeod, S. E. Mbanefo, Mary Motton, Ann C. Parry, D. S. Paton, Ruth E. Pawlyn, G. F. Pinckney, Jean M. H. Pinkerton, R. D. Stride, Denovah Vardy.

M.B., Ch.B.—*Second Examination :* Rosalind P. Adams, Joan H. Barnes, B. B. Beeson, Joan K. Cheeseman, Jennifer M. Comely, Diana F. J. Duncan, F. V. Eastman, A. R. Ebanks, P. C. Farrant, Katharine E. Fawcus, Elizabeth M. Fraser, D. M. Gambier, Patricia M. Gilbert, J. C. Gregg, Pamela Hawkins, Pamela M. Hellings, G. C. K. Herapath, Sybil E. Jacob, G. Lucas, Ellen M. Marron, J. P. Martin, Elizabeth M. Morgan, Jean Musgrave, P. A. Normandale, Margaret R. Salisbury, H. Schnieden, J. D. Sheward, June M. Smith, Medora Storkey, G. Walters, Olwen J. West, Catherine C. White.

M.B., Ch.B.—*Final Examination :* Kathleen Blott, W. H. K. Carpenter, Pamela M. Farmer, H. W. Jones, Florence E. Moore (*a*), H. S. Paull, C. D. R. Pengelly, Dorothy E. M. Pierce, A. D. Stewart, K. D. J. Vowles (*b*). *Section I only :* Patricia M. Appleton, M. J. Dunn, N. G. Glen, W. H. N. Moore, Josephine C. Mulcahy, G. Winternitz.

B.D.S.—*First Examination :* M. Bernstein, A. H. Chivers, M. N. Simmonds, Ruth A. Yearn.

B.D.S.—*Second Examination :* D. C. Berry, J. S. Cooper.

B.D.S.—*Third Examination :* L. M. Lloyd.

B.D.S.—*Final Examination :* G. A. J. Comyn, A. N. R. Gibson, A. F. J. Smith.

L.D.S.—*First Examination :* G. D. Everard.

L.D.S.—*Second Examination :* J. P. B. Pengelly, J. T. Sanders.

L.D.S.—*Third Examination :* M. J. Bartlett, Margaret J. Webb.

L.D.S.—*Final Examination :* L. G. D. Brown, J. C. Nicholas.

(*a*) With Distinction in Obstetrics.

(*b*) With Distinction in Surgery and in Public Health.

